# AUTOLOGOUS SERUM SKIN TEST IN CHILDREN

**DOI:** 10.4103/0019-5154.39576

**Published:** 2008

**Authors:** Kiran V Godse

**Affiliations:** *From Shree Skin Centre and Laboratory, 21/22, L market, Sector 8, Nerul, Navi Mumbai - 400 706, India*

**Keywords:** *Children*, *autoimmune urticaria*, *autologous*, *serum*

## Abstract

Chronic urticaria in children occurs less often than adults. Out of 17 children (age group 6-16 years, Table 2), six children (2 boys and 4 girls, 35%) showed a positive result in form of wheal and flare more than 1.5 mm than saline control. Autologous serum skin test is cheap, is easy to perform, and, if performed as appropriate, it has good sensitivity and even better specificity at detecting autoantibodies in children.

## Introduction

Chronic urticaria (CU) in children occurs less frequently than that for adults. While acute urticaria in children can be symptomatically treated without further investigations, CU requires a search for trigger factors. Autoimmune urticaria can occasionally be documented in children.^1^ It is known that the binding of an antigen (allergen) to antigen-specific IgE on mast cells and basophils causes cell degranulation, resulting in the release of histamine and other vasoactive mediators that are responsible for the clinical symptoms. However, most patients with CU have no specific allergic trigger for mast cell or basophil activation, and when no cause can be identified, the final diagnosis is chronic idiopathic urticaria (CIU).^2^ One-third of the patients with CIU have circulating functional autoantibodies against the high affinity IgE receptor FcepsilonRI or IgE. The intradermal injection of autologous serum causes a wheal-and-flare reaction in many patients with CIU and this reaction forms the basis of the autologous serum skin test (ASST).^3^ There are few reports of FcεRI autoantibodies in children with CU.^4^,^5^ This study was done to assess the incidence of AU in children in India. One study from India found positive ASST in 27% adults.^6^

## Materials and Methods

This study included 17 children (8 boys and 9 girls) in the age group of 6-16 years (mean age: 10.9 years) with CU of more than a duration of 6 weeks' duration. In all cases, questions regarding food allergies, drug intake, signs of infection, causes of physical urticaria, insect bites and personal and family history of atopy were asked. The clinical characteristics of the disease, such as duration, recurrence and associated angioedema and symptoms of anaphylaxis were also investigated. Children with predominant physical urticaria were not included in the study. Routine investigations such as complete blood count and urine examination were done to rule out the focus of infection. Advanced tests for autoimmune diseases were not done due to high cost of investigations. None of the children were on treatment other than antihistamines for urticaria. Antihistamines included hydroxyzine and cetirizine in all patients. The complete history was obtained and physical examination was done to rule out the systemic diseases. All children were advised to stop antihistamines for 3 days. Parents were informed about the nature of the test. The test was performed when urticaria was active. Blood was collected in a sterile, plain Vacutainer and centrifuged for 10 min at 2500 rpm. Serum was separated and intradermally injected (0.05 ml) on the left forearm with sterile tuberculin syringe. Sterile normal saline (0.05 ml) was intradermally injected few centimeters away from test site. Reading was taken at 30 min for presence of wheal and flare with the help of a magnifying lens and scale. According to Sabroe, a positive test indicates the presence of wheal and flare for more than 1.5 mm diameter than that of saline control.^7^

## Results

**Fig. 1:**
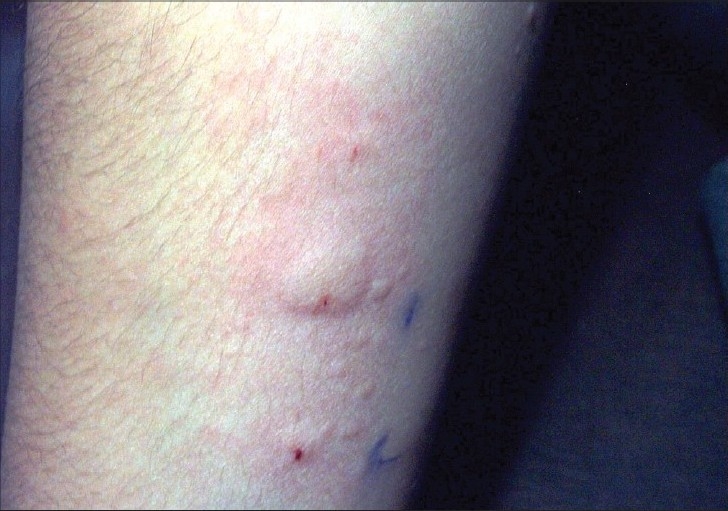
Positive ASST with incidental wheals

Out of 17 children (age group: 6-16 years, Table 1), 6 children (2 boys and 4 girls, 35%) showed a positive result in form of wheal and flare to be more than 1.5 mm than saline control. One child had strongly positive ASST (test wheal more than 3 times saline wheal) (Fig. 1). Complete blood count showed the hemoglobin to be less than 10 gm% in four children and leucocytosis in three children. Eosinophilia was present in one child. Urine examination did not reveal abnormal findings. Urticaria was present for 2-6 months in most children (Table 2). The history of angioedema was present in five children. Family history of atopy was present in four children.

**Table 1:** Age group categorization of patients with urticaria

Age group of children (years)	No. of boys	No. of girls
6-9	3	2
10-12	4	4
13-16	1	3

**Table 2:** Number of patients as per duration of urticaria

Duration of urticaria	2-4 months	More than 4 months
No. of children	13 (7 boys and 6 girls)	4 (1 boy and 3 girls)

Urticaria is a common disease in children. In contrast to the ease of its diagnosis, etiologic factors are often difficult to determine. Chronic urticaria is defined as the daily or almost daily occurrence of short-lived wheals for at least 6 weeks. Chronic urticaria in children is less commonly reported. The natural history of urticaria in children is distinct from that in adults.

A study from tertiary referral centre from India reported that 80% children had CU out of 44 children.^8^ Few studies in children showed that in more than 70% of cases, exact cause could not be identified.^9^,^10^ In the Western literature, a positive ASST has been reported in 25-45% of adult patients with CIU.^11^ A positive test is suggestive but not the diagnostic of an autoimmune basis for the patient's urticaria. Confirmation is needed by *in vitro* testing of the patient's serum for anti-FcεR1 or anti-IgE autoantibodies.^11^ A review of 94 children with CU revealed that 58% became symptom-free for 6 months or more, whereas the cause in this study was determined in only 16%.^12^ In another report of 226 children (age: 1-14 years) with CU, only 21% were determined to have the causal factor.^9^

A case report from Italy reported a 6-year-old child with strong skin reactivity upon the intradermal injection of autologous serum suggested an autoreactive pathogenesis; however, the patient's serum could not induce histamine release from basophils *in-vitro*, indicating the presence of a histamine-releasing factor that is specific for mast cells - possibly other than an anti-FcepsilonRI or anti-IgE antibody.^13^ The autologous serum skin test has been proposed as a surrogate test to define the presence of these autoantibodies, although it identifies the presence of histamine-releasing factor, not necessarily the antibody.^14^

Autologous serum skin test is cheap, easy to perform and if performed properly,^3^ it shows good sensitivity and even better specificity in (Fig. 1) detecting autoantibodies even in children; therefore, it can be used as a predictive clinical test to diagnose AU, particularly in places where the basophil histamine-releasing test is not available. This test is not available in India.

We found 35% children positive with ASST that is indicative of AU. A large-scale study from Italy showed 45% positive ASST (22 out of 49 children). Serum-induced basophil histamine release was positive in 16 out of 31 children (52%) with CIU in the same study.^5^ A study from South Africa found Anti-FcepsilonRIalpha autoantibodies positive in 37 (47%) out of 78 children with CU and in none of 33 with atopic eczema dermatitis syndrome.^15^ Intradermal test with autologous serum may be useful in revealing the autoreactive nature of CU, thereby avoiding a frustrating search for other causes of the disease. In children presenting with chronic or recurrent urticaria, the diagnostic workup should include the autologous serum skin test.

We hope that ASST will help the dermatologist to diagnose a child with AU and use immunosuppressive therapies when routine treatment fail to show control of urticaria. The significance of positive ASST in children must be investigated with advanced tests and large studies.
